# Supplementary material for: A Robust Assay to Monitor Ataxin-3 Amyloid Fibril Assembly
Source: Cells. 2022 Jun 19;11(12):1969. doi: 10.3390/cells11121969 (PMC9222203; doi:10.3390/cells11121969)
Supplement: Supplementary file 1 [file cells-11-01969-s001.zip › cells-1743016-SM.pdf]

Supporting Information

A robust assay to monitor ataxin-3 amyloid fibril assembly

Francisco Figueiredo[1,2,3,4], Mónica Lopes-Marques[1,2,5], Bruno Almeida[6,7], Nena Matscheko[8], Pedro Martins[1,2], Alexandra Silva[1,2*], Sandra Macedo-Ribeiro[1,2*]

[1]Instituto de Investigação e Inovação em Saúde (i3S), Universidade do Porto, Porto, Portugal;

[2]Instituto de Biologia Molecular e Celular (IBMC), Universidade do Porto, Porto, Portugal;

[3]International Iberian Nanotechnology Laboratory (INL), Braga, Portugal

[4]Instituto de Ciências Biomédicas Abel Salazar (ICBAS), Universidade do Porto, Porto, Portugal

[5]Department of Biology, Faculty of Sciences, University of Porto, Porto, Portugal

[6]Life and Health Sciences Research Institute (ICVS), School of Medicine, University of Minho, Braga, Portugal;

[7]ICVS/3B's—PT Government Associate Laboratory Braga, Portugal

[8]Dynamic Biosensors GmbH, Martinsried, Germany;

[*]Correspondence: sribeiro@ibmc.up.pt (S.M-R.), a.silva@ibmc.up.pt (A.S.)

S1. Primers and Plasmid Sequences

Supplementary Table S1. Nucleotide sequences of primers and synthetic genes used in this work.

Name	Sequence	Purpose
I77K_For	5´-gatgacagtggtttttctctaaacaggttataagcaatgccttg-3´	Mutagenesis of Atx3 13Q
I77K_Rev	5´- caaggcattgcttataacctgtttagagaaaaaccact gtcatc-3´	Mutagenesis of Atx3 13Q
Q78K_For	5’-gatgacagtggtttttctctattaaggttataagcaatgccttg-3´	Mutagenesis of Atx3 13Q
Q78K_Rev	5´-caaggcattgcttataaccttaatagagaaaaaccactgtcatc-3´	Mutagenesis of Atx3 13Q
W87K_For	5’-gcaatgccttgaaagttaagggtttagaactaatcctgttcaac-3’	Mutagenesis of Atx3 13Q
W87K_Rev	5’-gttgaacaggattagttctaaacccttaactttcaaggcattgc-3’	Mutagenesis of Atx3 13Q
R388G_For	5’-tggcctgacagatctccctgttgttgttgctgc-3’	Mutagenesis of Atx3 77Q
R388G_Rev	5’-gcagcaacaacaacagggagatctgtcaggcca-3’	Mutagenesis of Atx3 77Q
77Q_opt	5’-<br>atgcatcgaccaaaactgattggcgaagaactggctcaactgaaagaacagcgtgtgcataagaccgacctggaacgtgtcctggaagcaaatgacggcagcggcatgctggatga<br>agacgaagaagatctgcagcgtgccctggcactgtctcgtcaggaaattgatatggaagacgaagaagcagatctgcgtcgcgctattcagctgtcaatgcagggcagctctcgtaac<br>atctcgcaggacatgacccagacgagcggtaccaatctgacgtctgaagaactgcgcaaacgtcgcgaagcatattttgaaaaacagcaacagaagcaacaacagcagcaacaac<br>aacaacaacagcagcagcagcagcaacaacaacagcaacaacaacagcaacagcagcaacagcaacagcaacagcagcaacaacagcaacagcaacaacagcaacagcaac<br>aacagcaacaacagcaacaacagcaacaacagcaacaacagcaacaacagcaacagcaacaacagcagcagcaacagcagcaacaacaacagagagatctgtcaggccagag<br>ttcccatccgtgtgaacgtccggccacctcaagcggtgcactgggtagtgatctgggtgacgccatgtccgaagaagacatgctgcaggcagcagtgacgatgtccctggaaaccgtg<br>cgtaacgacctgaaaaccgaaggcaaaaaataagacccagctttcttgtaca-3’	Synthetic sequence of codon-optimized construct for production of Atx3(77Q)

13Q_opt	5’-<br>atgcatcgaccaaaacttattggagaagaattagcacaactaaaagagcaaagagtccataaaacagacctggaacgagtgttagaagcaaatgatggctcaggaatgttagacgaagatgaggaggatttgcagagggctctggcactaagtcgccaagaaattgacatggaagatgaggaagcagatctccgcagggctattcagctaagtatgcaaggtagttccagaaacatatctcaagatatgacacagacatcaggtacaaatcttacttcagaagagcttcggaagagacgagaagcctactttgaaaaacagcagcaaaagcagcaacagcagcagcagcagcagcagcaggggggacctatcaggacagagttcacatccatgtgaaaggccagccaccagttcaggagcacttgggagtgatctaggtgatgctatgagtgaagaagacatgcttcaggcagctgtgaccatgtctttagaaactgtcagaaatgatttgaaaacagaaggaaaaaaataagacccagctttcttgtaca-3’	Synthetic sequence of codon-optimized construct for production of Atx3(13Q)

Supplementary Table S2. Amino acid sequences of proteins and peptides used in the aggregation assays. Josephin Domain (Green); Aggregation-prone Region (Red); polyQ (Orange); Atx3 77Q mutants (Blue, Bold); Atx3 13Q ubiquitin binding site 2 mutants (Purple, Bold).

**Name**	**Sequence**	**Extinction Coefficients ($M^{-1}.cm^{-1}$)**
Atx3 JD	MSYYHHHHHHLESTSLYKKAGWMESIFHEKQEGSLCAQHCLNNLLQGEYFSPVELSSIAHQLDEEERMRMAEGGVTSEDYRTFLQQPSGNMDDSGFFSIQVISNALKVWGLELILFNSPEYQRLRIDPINERSFICNYKEHWFTVRKLGKQWFNLNSLLTGPELISDTYLALFLAQLQQEGYSIFVVKGDLPDCEADQLLQMIR	35660
Atx3 D1	MSYYHHHHHHLENLYFQGMESIFHEKQEGSLCAQHCLNNLLQGEYFSPVELSSIAHQLDEEERMRMAEGGVTSEDYRTFLQQPSGNMDDSGFFSIQVISNALKVWGLELILFNSPEYQRLRIDPINERSFICNYKEHWFTVRKLGKQWFNLNSLLTGPELISDTYLALFLAQLQQEGYSIFVVKGDLPDCEADQLLQMIRVQQMHRPKLIGEELAQLKEQRVHKTDLERVLEANDGSGMLDEDEEDLQRALALSRQEIDMEDEEADLRRAIQLSMQGSSR	30160
Atx3 13Q	MSYYHHHHHHLENLYFQGMESIFHEKQEGSLCAQHCLNNLLQGEYFSPVELSSIAHQLDEEERMRMAEGGVTSEDYRTFLQQPSGNMDDSGFFSIQVISNALKVWGLELILFNSPEYQRLRIDPINERSFICNYKEHWFTVRKLGKQWFNLNSLLTGPELISDTYLALFLAQLQQEGYSIFVVKGDLPDCEADQLLQMIRVQQMHRPKLIGEELAQLKEQRVHKTDLERVLEANDGSGMLDEDEEDLQRALALSRQEIDMEDEEADLRRAIQLSMQGSSRNISQDMTQTSGTNLTSEELRKRREAYFEKQQQKQQQQQQQQQQGDLSGQSSHPCERPATSSGALGSDLGDAMSEEDMLQAAVTMSLETVRNDLKTEGKK	31650
Atx3 77Q	MSYYHHHHHHLENLYFQGMESIFHEKQEGSLCAQHCLNNLLQGEYFSPVELSSIAHQLDEEERMRMAEGGVTSEDYRTFLQQPSGNMDDSGFFSIQVISNALKVWGLELILFNSPEYQRLRIDPINERSFICNYKEHWFTVRKLGKQWFNLNSLLTGPELISDTYLALFLAQLQQEGYSIFVVKGDLPDCEADQLLQMIRVQQMHRPKLIGEELAQLKEQRVHKTDLERVLEANDGSGMLDEDEEDLQRALALSRQEIDMEDEEADLRRAIQLSMQGSSRNISQDMTQTSGTNLTSEELRKRREAYFEKQQQKQQQQQQQQQQQQQQQQQQQQQQQQQQQQQQQQQQQQQQQQQQQQQQQQQQQQQQQQQQQQQQQQQQQQQQQQQQRDLSGQSSHPCERPATSSGALGSDLGDAMSEEDMLQAAVTMSLETVRNDLKTEGKK	31650
Atx3 77Q R388G	MSYYHHHHHHLENLYFQGMESIFHEKQEGSLCAQHCLNNLLQGEYFSPVELSSIAHQLDEEERMRMAEGGVTSEDYRTFLQQPSGNMDDSGFFSIQVISNALKVWGLELILFNSPEYQRLRIDPINERSFICNYKEHWFTVRKLGKQWFNLNSLLTGPELISDTYLALFLAQLQQEGYSIFVVKGDLPDCEADQLLQMIRVQQMHRPKLIGEELAQLKEQRVHKTDLERVLEANDGSGMLDEDEEDLQRALALSRQEIDMEDEEADLRRAIQLSMQGSSRNISQDMTQTSGTNLTSEELRKRREAYFEKQQQKQQQQQQQQQQQQQQQQQQQQQQQQQQQQQQQQQQQQQQQQQQQQQQQQQQQQQQQQQQQQQQQQQQQQQQQQQQ**G**DLSGQSSHPCERPATSSGALGSDLGDAMSEEDMLQAAVTMSLETVRNDLKTEGKK	31650
Atx3 13Q I77K Q78K W87K	MSYYHHHHHHLENLYFQGMESIFHEKQEGSLCAQHCLNNLLQGEYFSPVELSSIAHQLDEEERMRMAEGGVTSEDYRTFLQQPSGNMDDSGFFS**KK**VISNALKV**K**GLELILFNSPEYQRLRIDPINERSFICNYKEHWFTVRKLGKQWFNLNSLLTGPELISDTYLALFLAQLQQEGYSIFVVKGDLPDCEADQLLQMIRVQQMHRPKLIGEELAQLKEQRVHKTDLERVLEANDGSGMLDEDEEDLQRALALSRQEIDMEDEEADLRRAIQLSMQGSSRNISQDMTQTSGTNLTSEELRKRREAYFEKQQQKQQQQQQQQQQGDLSGQSSHPCERPATSSGALGSDLGDAMSEEDMLQAAVTMSLETVRNDLKTEGKK	26150

HsUBB3	MQIFVKTLTGKTITLEVEPSDTIENVKAKIQDKEGIPPDQQRLIFAGKQLEDGRTLSDYNIQKESTLHLVLRLRGGMQIFVKTLTGKTITLEVEPSDTIENVKAKIQDKEGIPPDQQRLIFAGKQLEDGRTLSDYNIQKESTLHLVLRLRGGMQIFVKTLTGKTITLEVEPSDTIENVKAKIQDKEGIPPDQQRLIFAGKQLEDGRTLSDYNIQKESTLHLVLRLRGGC	4470
QBP1	SNWKWWPGIFD	16500

S2. Step by Step Protocols

S2.1 Ataxin-3 Expression

The full list of reagents used in this work is detailed in section 4 (S4) of the Supporting Information.

1. First, *E. coli* BL21(DE3)-SI (Life Technologies, Carlsbad, CA, USA) was transformed with the plasmid carrying the selected ataxin-3 variant by adding 1 µL of selected plasmid (40-100 ng μL^{-1}) to 50 µL of competent cells in an microtube, and incubating for 30 min on ice.

2. BL21(DE3)-SI cells were heat-shocked for 45 seconds at 42°C, followed by 10 min incubation on ice. Next, 500 µL LB medium without NaCl (LB_{ON}) (Formedium, UK) were added and the cells were incubated for 1 h at 37 °C and 180 rpm.

3. BL21(DE3)-SI cells were pelleted by centrifugation for 1 min at 11,200 g, and the supernatant was removed. Cells were gently resuspended in 100 µL fresh LB_{ON}.

4. The suspension was plated on a LB_{ON} plate supplemented with 100 µg mL^{-1} ampicillin and the plate incubated overnight at 37 °C.

5. The starter culture was prepared in a 500 mL baffled Erlenmeyer containing 150 mL LB_{ON} supplemented with 100 µg mL^{-1} ampicillin. Next 4/5 isolated colonies selected from the stored plate were used to inoculate the culture. The starter culture was incubated overnight at 37 °C and 180 rpm.

6. The culture was expanded by inoculating 20 mL of starter culture into a 2 L baffled Erlenmeyer containing 500 mL LB_{ON} supplemented with 100 µg mL^{-1} ampicillin and 0.4 % (w/v) glucose. The culture was maintained at 37 °C and 180 rpm, and OD_{600} was checked regularly.

7. When culture OD_{600nm} reached 0.8, the Erlenmeyer was cooled to 30 °C for 30 min with no shaking (1 mL sample was retrieved for SDS-PAGE analysis to assess ataxin-3 expression before induction).

8. Protein expression was induced by adding 50 mL of induction medium (2x LB_{ON} with 3 M NaCl) to the Erlenmeyer to achieve the final concentration of 273 M NaCl.

Protein expression proceeded at 30 °C and 180 rpm for 3 h (a 1 mL sample was retrieved for SDS-PAGE analysis to assess ataxin-3 expression after induction).

9. Cells were harvested by centrifugation for 20 min at 3,993 g at 4 °C. The supernatant was discarded and the cell pellet was carefully resuspended (20 mL per L of cell culture) in ice-cold Buffer A (20 mM sodium phosphate pH 7.5, 500 mM NaCl, 2.5% (v/v) glycerol, 20 mM imidazole) supplemented with 100 µg L^{-1} lysozyme. Cell suspensions were stored at -20 °C in 50 mL disposable screw cap tubes until further use.

S2.2 Ataxin-3 Purification

1. The cell suspension was slowly thawed by placing the 50 mL tube under running tap water for 15 min. The cell suspension was transferred to a 250 mL Erlenmeyer and the cells were disrupted by slowly stirring for 1 h on ice in the presence of lysozyme (0.02 mg mL^{-1}), DNAse (0.02 mg mL^{-1}), RNAse (0.02 mg mL^{-1}), $MgCl_2$ (1 mM) and PMSF (1 mM). After cell disruption, 10 µL of the sample was collected for SDS-PAGE analysis.
2. The cell lysate was centrifuged for 45 min at 34,864 g at 4 °C. The supernatant was filtered (0.45 µm low protein binding filter - Whatman Puradisc 25, ref: 6747-2504) and loaded onto a Ni^{2+}-charged IMAC HiTrap column (GE Healthcare Life Sciences, ref: 17-0920-05) pre-equilibrated with 20 column volumes (CV) of Buffer A.
3. Bound proteins were eluted with 10 CV steps of increasing imidazole concentration (50, 250 and 500 mM imidazole in Buffer A; Supplementary Figure S1A-D). Ataxin-3-containing fractions eluted with 250 mM imidazole were supplemented with EDTA to a final concentration of 1 mM. The collected fractions (5-10 mL) were carefully homogenised, filtered (0.22 µm low protein binding filter, Millipore, ref: SLGV033RB) and kept on ice.
4. Monomeric ataxin-3 was obtained following purification by size exclusion chromatography (SEC) using a HiPrep 20/60 Sephacryl S-300 HR column (GE Healthcare Life Sciences, ref: 17-1196-01) equilibrated in SEC buffer (20 mM sodium phosphate pH 7.5, 150 mM NaCl, 5 % (v/v) glycerol, 2 mM EDTA, 1 mM DTT; Supplementary Figure S1A-D). The fractions (2.5 mL) corresponding to the central part of the peak of monomeric ataxin-3 were collected (Supplementary Figure S1A-D) and kept on ice prior to concentration in a centrifugal ultrafiltration device (Amicon Ultra-15, 10 kDa molecular weight cut-off, Millipore) at 4 °C. Final protein concentrations ranged

between 10 and 20 mg mL^{-1} and were calculated using the extinction coefficients detailed in Table S1 from the sample absorbance at 280 nm measured in a Nanodrop 1000 spectrophotometer (Thermo Fisher Scientific).

5. The concentrated protein was transferred into a low protein binding microtube (Eppendorf, ref: 022431081), centrifuged for 10 min at 16,363 g at 4 °C to remove putative aggregates. The supernatant was transferred into a new low protein binding microtube, homogenized and the final concentration was determined from the absorbance at 280 nm, as detailed in step 4, above.

6. Purified ataxin-3 proteins were stored at -80 °C in low protein binding microtubes in 50 µL or 100 µL aliquots, following flash freezing in liquid nitrogen.

S2.3 Ataxin-3 Aggregation Assay / Thioflavin-T Aggregation Assay

1. Purified ataxin-3 aliquots were thawed on ice, centrifuged for 10 min at 16,363 g at 4 °C, filtered in a low protein-binding filter (0.22 µm; Millipore, ref: UFC30GV00) and injected on a Superose 12 10/300 GL (GE Healthcare Life Sciences, ref: 17-5173-01) column equilibrated in aggregation buffer (20 mM HEPES pH 7.5, 150 mM NaCl, 1 mM DTT or 20 mM Sodium phosphate pH 7.5, 150 mM NaCl, 1 mM DTT) (Figure 1D). Fractions (0.4 mL) corresponding to the central part of the lower molecular weight peak (Figure 1D) were collected, and the protein concentration was determined as described in step 4 of S2.2, above. The final concentration of protein was adjusted to 15 µM in aggregation buffer.

2. The 3.5 M (in water) stock solution of Thioflavin-T (ThT) was pre-diluted to 500 µM in aggregation buffer.

3. A master mix (300 µL, i.e., enough to perform 5 replicates of 50 µL each), was prepared, with final concentrations of 5 µM and 30 µM for ataxin-3 and ThT, respectively. Any compound or protein being tested was added to the master mix in this step, regardless of the buffer/solvent it was in. Pre-dilution was made in the aggregation buffer. The master mix was gently mixed to avoid the formation of air bubbles.

4. Fifty µL master mix were pipetted to each microplate well (Thermowell 96-Well Polycarbonate PCR Microplates, Costar, Tewkesbury, MA, USA or 384-well low flange, black, flat bottom, polystyrene microplate, Corning, Kennebunk, ME). Air bubbles were removed by centrifuging the plate for 2 min at 306 g at RT.

5. To avoid evaporation during the aggregation assay, 20 µL of paraffin oil were added to each reaction well prior to transferring the plate to the reader, previously heated to 37 °C.

6. ThT fluorescence was measured at 37 °C on a HIDEX CHAMELEON V plate reader (Turku, Finland) or a FluoDia T70 microplate fluorimeter (Photon Technology International, Edison, NJ, USA) at 480 nm (440 nm excitation) every 30 min over a period of 60 h. Plates were shaken for 3 seconds before each set of readings.

7. After 60 h, the assay was stopped and the data were analysed with Prism 9 software (GraphPad Software, San Diego, CA, USA).

S2.4 Transmission Electron Microscopy Assay

This protocol was adapted from Rames *et al.*, 2014 [1].

S2.4.1 Preparation of Fresh Negative Staining Solution at 1% (w/v)

1. One mg uranyl acetate (UA) powder was added to a glass bottle containing 100 mL deionized water in a dark room or box.

2. The bottle was wrapped in aluminium foil to protect the solution from light and the solution was stirred overnight (ON) at RT in a dark room/box.

3. The solution was gently filtered through a 0.2 µm syringe filter (Puradisc FP 30mm, Whatman), using a 5mL syringe wrapped in aluminium foil to prevent exposure to light and the filtered solution was collected in 2 mL aliquots in microtubes also wrapped in aluminium foil.

4. The negative staining solution aliquots were frozen in liquid nitrogen immediately after filtration and stored at -80 °C until further use.

S2.4.2 Preparation of Negative Staining Workstation and Incubation Workstation

5. A vial of 1 % (w/v) UA solution was thawed in a water bath at RT with the aluminium foil wrapped around the vial to prevent exposure to light.

6. The solution was filtered using a 0.2 µm filter on a 1 mL syringe wrapped in aluminium foil. The filtered solution was collected into a new aluminium foil-wrapped vial, and placed inside a covered ice box until use.

7. Solution plates were made by finger pushing a long enough piece of Parafilm sheet onto the surface of an empty 200 µL tip box cover plate. This generated rows of circular depressions with a diameter of approximately 5 mm. Six depressions were made in each row and the 200 µL tip box cover plate was placed on a surface of flattened ice inside an ice container, as a staining workstation.

8. For each grid, a row was pipetted consisting of 3 consecutive droplets of 35 µL deionized water followed by 3 consecutive droplets of 35 µL filtered UA solution. The staining workstation was covered with a lid to minimize exposure to light.

9. An Electron Microscopy (EM) grid incubation station was prepared by filling a box (e.g. an empty pipet tip container) halfway with ice, placed next to a hanger prepared to hold the sample tweezers. The sample tweezers were placed on the hanger, so that the tweezers' tips were nearby the ice surface and partly shielded by the box plastic cover.

S2.4.3 – Negative-Staining Operation

10. Thin carbon film-coated EM grids were glow-discharged for 10 sec.

11. Each grid was picked up with tweezers, which were hooked into the hanger so that the grid was kept at a 45° tilt angle and close to the ice surface inside the incubation station.

12. Five µl of protein sample (diluted or not, depending on the nature of the sample) were deposited (immediately after dilution, when performed) on the carbon side of the EM grid.

13. The sample was incubated for 1 min on the EM grids inside the incubation station.

14. Excess solution was removed by quickly touching the grid edge with filter paper.

15. The grid was quickly placed in the surface of the first drop of water atop the Parafilm sheet and the excess water was promptly removed with filter paper.

16. The EM grid was washed two more times on the remaining two water droplets on the staining workstation by repeating Step 15 (Step 16 can be skipped for very water sensitive samples).

17. The EM grid was floated immediately on the surface of the first drop of UA solution, right after the excess water on the EM grid was removed, and incubated for 10 sec. The washing procedures must be finished within 3 seconds before floating the grid on the surface of the UA solution. Shorter washing times lead to better quality.

18. The tweezers were cleaned by piercing filter paper 2-3 times with the tips.

19. Excess solution was removed from the grid by touching its edge with filter paper, and then the grid was floated on the second drop of UA.

20. Steps 18 and 19 were repeated for the third droplet of UA solution and the staining station was covered for 1 min.

21. Excess solution was removed by blotting the back (opposite to the carbon side) of the grid with filter paper.

22. The grid was placed on a sheet of filter paper inside a partially covered petri dish, and allowed to dry for at least 30 min at RT. The grid was stored in a TEM grid box until examination.

S2.5 Thermal Shift Assay Protocol

The melting temperatures of ataxin-3 variants were determined using the hydrophobic fluorescent dye SYPRO Orange. For each condition, 12.5 µL 10x SYPRO Orange (Invitrogen; diluted in ataxin-3 SEC buffer from 5000x stock) were mixed with 12.5 µL ataxin-3 (0.8 mg mL^{-1}) and loaded in a white 96-well PCR plate (Bio-Rad). Three replicates were prepared for each condition. The thermal shift assay was performed in an iCycler iQ5 Multicolor Real Time PCR detection system (Bio-Rad) running the following protocol: heating from 25 °C to 85 °C with a 30s hold time every 0.5 °C, followed by a fluorescence reading using Cy3 dye filter (excitation/emission, 545/585). Melting curves were analysed using the CFX Manager software (Bio-Rad) to calculate melting temperature (Tm) from the minimum value of the first derivative curve of the melting curve.

S3. Supporting Figures

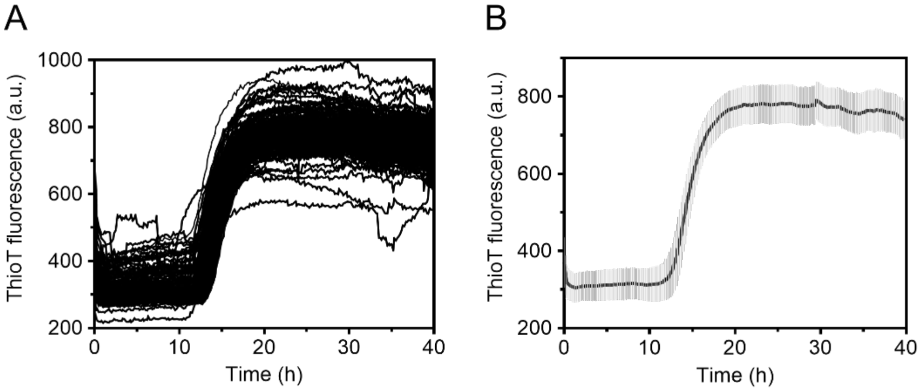

Supplementary Figure S1. Repeatability of the Atx3 13Q aggregation assay in HEPES buffer. (A) 192 replicas of Atx3 13Q (3 µM) ThT assay using the HIDEX CHAMELEON V plate reader. **(B)** Mean and standard deviation of the 192 replicates presented in (A).

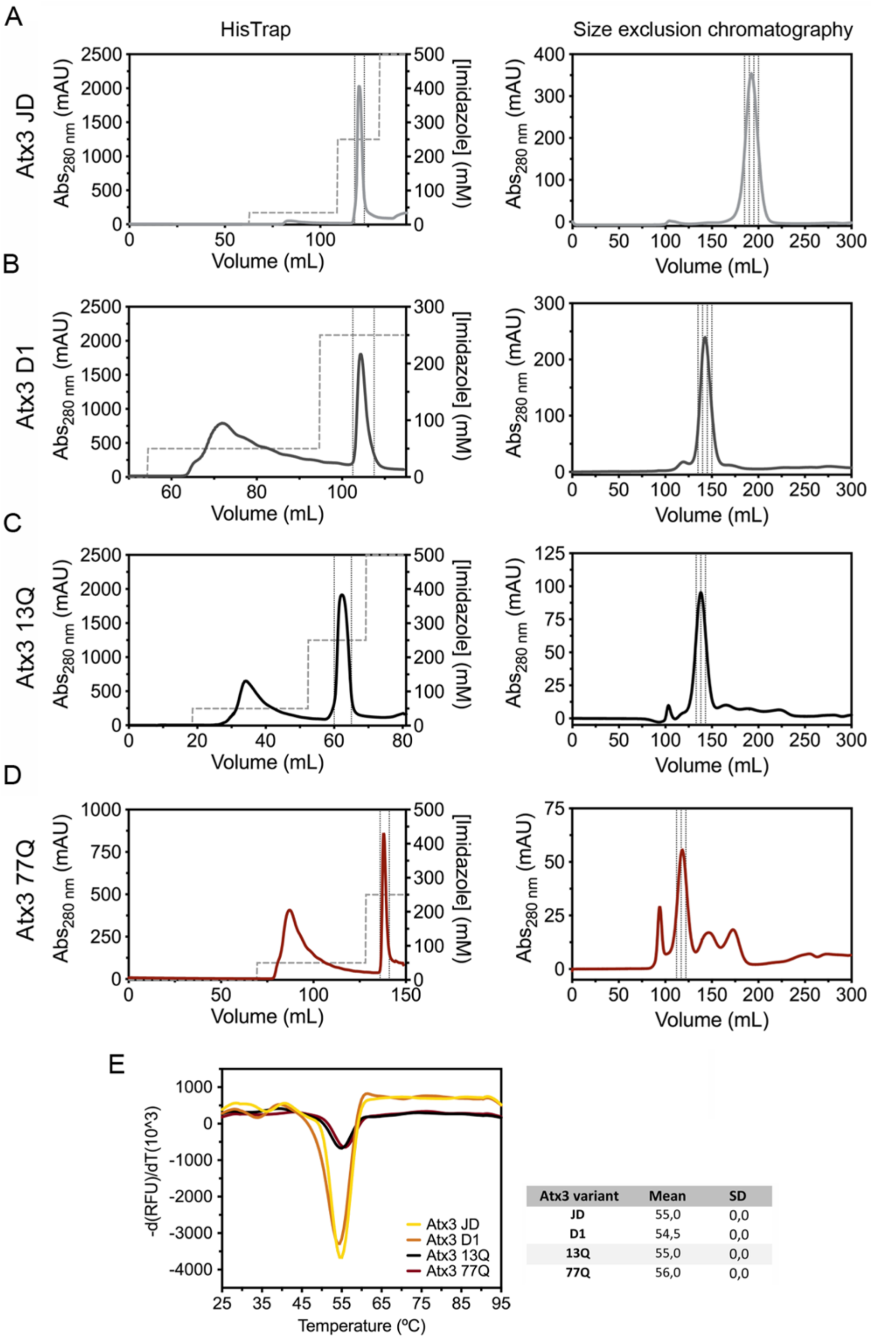

Atx3 variant	Mean	SD
JD	55,0	0,0
D1	54,5	0,0
13Q	55,0	0,0
77Q	56,0	0,0

Supplementary Figure S2. Purification and thermal shift assay of different Ataxin-3 constructs. Chromatograms showing the purification of the ataxin-3 constructs **(A)** JD, **(B)** D1, **(C)** Atx3 13Q and **(D)** Atx3 77Q on Ni^{2+}-charged IMAC HiTrap (left) and HiPrep 20/60 Sephacryl S-300 HR (right) columns. Grey dashed lines represent imidazole concentration and grey solid lines indicate fractions selected for further purification or storage. **(E)** Thermal shift assay of Atx3 JD, Atx3 D1, Atx3 13Q and Atx3 77Q. Curves represent an average of 3 replicates per condition.

Population	Allele C	Allele G
African	0,831	0,169
American	0,816	0,184
East Asian	0,576	0,424
European	0,728	0,272
South Asian	0,663	0,337
Total	0,724	0,276

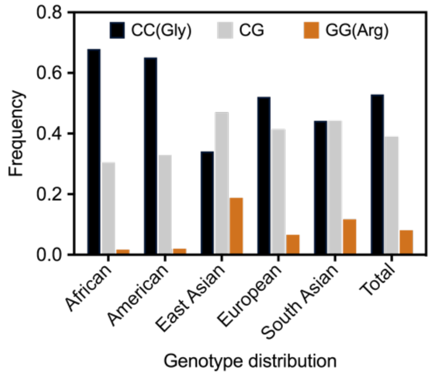

Supplementary Figure S3. Distribution of *Atxn3* variant rs12895357 in healthy population. Data collected from the 1000 Genome project Phase 3, which aggregated genomic information from 4973 healthy individuals, showed that homozygotic individuals for G306R are found in all populations being more prevalent in the East Asian population.

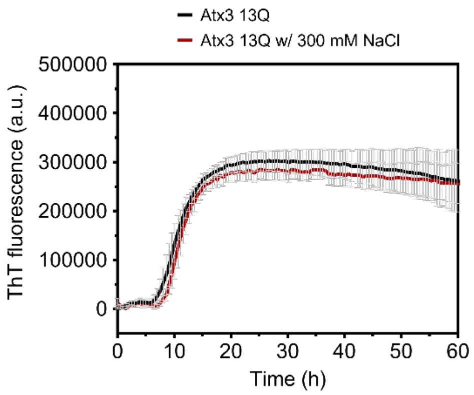

Supplementary Figure S4. Effect of 300 mM NaCl on ataxin-3 aggregation kinetics. ThT assay to measure the formation of amyloid-like species in Atx3 13Q in the presence of 300 mM NaCl. Curves represent the mean and standard deviation of five replicates for each condition using the FluoDia T70 microplate fluorimeter.

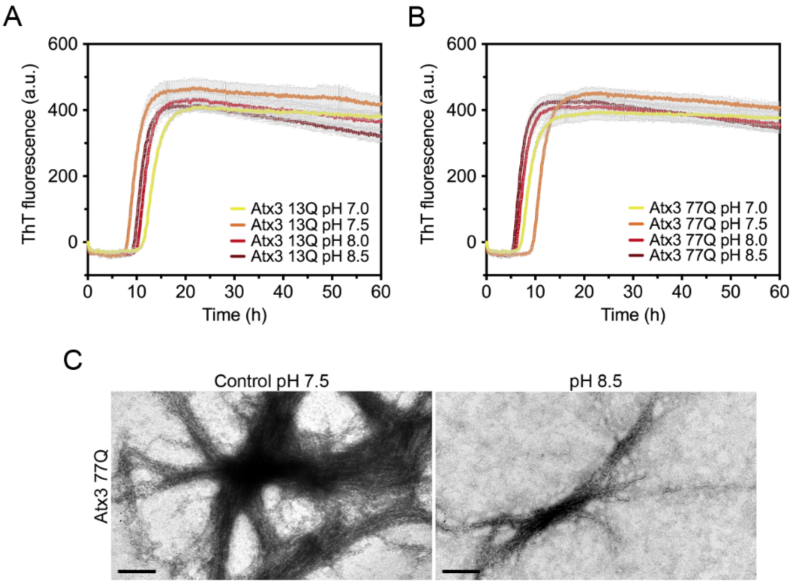

Supplementary Figure S5. Effect of pH on ataxin-3 aggregation kinetics. **(A**) Atx3 13Q and **(B)** Atx3 77Q amyloid-like formation, monitored by ThT fluorescence, at pH 7.0, 7.5 (control), 8.0 and 8.5. Curves represent the mean and standard deviation of five replicates for each condition using the HIDEX CHAMELEON V plate reader. **(C)** TEM images after negative staining of ThT assay endpoint samples (60 h, 37 °C) of Atx3 77Q at pH 7.5 (control) and 8.5. Scale bars correspond to 200 nm.

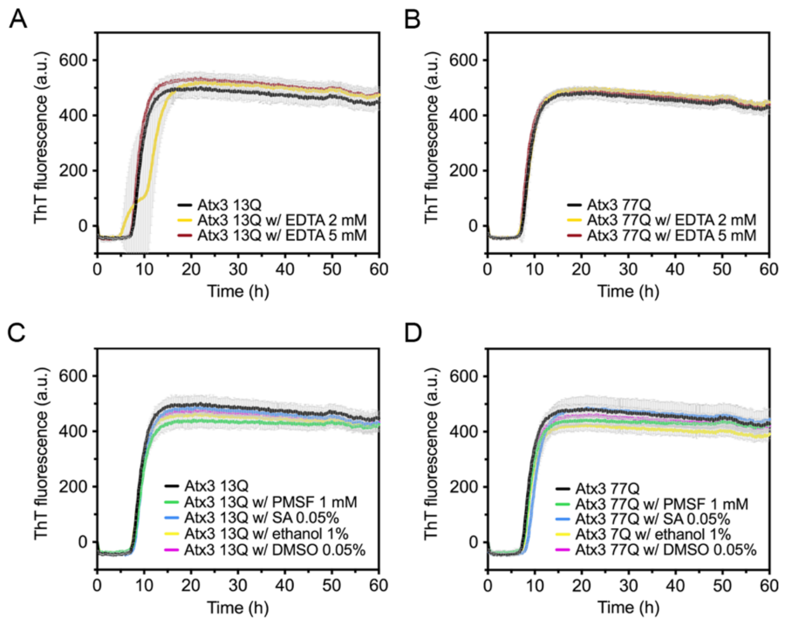

Supplementary Figure S6. Different aggregation buffer components do not affect ataxin-3 aggregation kinetics. ThT assay to measure the formation of amyloid-like species in Atx3 13Q **(A, C)** and Atx3 77Q **(B, D)** in the presence of different concentrations of EDTA, PMSF, sodium azide (SA), ethanol and DMSO. Curves represent the mean and standard deviation of five replicates for each condition using the HIDEX CHAMELEON V plate reader.

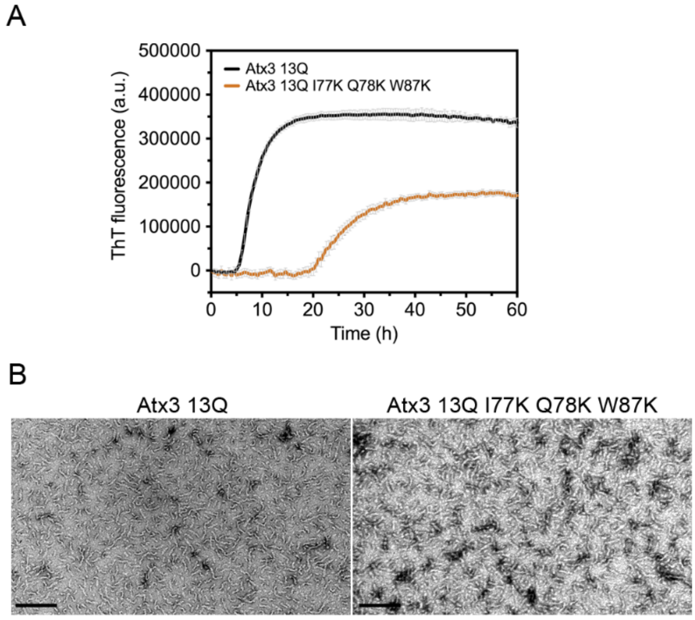

Supplementary Figure S7. Effect of mutating the second ubiquitin binding site of the JD on ataxin-3 aggregation kinetics. **(A)** ThT assay to measure the formation of amyloid-like species in Atx3 13Q and Atx3 13Q I77K Q78K W87K. Curves represent the mean and standard deviation of five replicates for each condition using a FluoDia T70 microplate fluorimeter. **(B)** TEM images after negative staining of ThT assay endpoint samples (60 h, 37 °C) of Atx3 13Q and Atx3 13Q I77K Q78K W87K. Scale bars correspond to 200 nm.

S4. Reagents Used

Supplementary Table S3. List of reagents used in this work.

Reagent	Brand	Reference
Acrylamide	Bio-Rad	1610148
Ampicillin	Formedium	AMP100
APS (Ammonium Persulfate)	Bio-Rad	161-0700
DNase	Sigma-Aldrich	DN25-100
DTT (Dithiothreitol)	Biosynth	D-8200
EDTA (Ethylenediaminetetraacetic acid)	Merck	1.08418.0250
Glucose	Millipore	1.04074.1000
Glycerol	VWR	24388.384
Glycine	Sigma-Aldrich	G8898
HEPES (4-(2-hydroxyethyl)-1-piperazineethanesulfonic acid)	Sigma-Aldrich	H3375
Imidazole	Acro Organics	301870010
IPTG (Isopropyl β-D-1-thiogalactopyranoside)	NZYTech	MB02603
Luria Broth w/o NaCl	Formedium	LBO0102
Lysozyme	Sigma-Aldrich	62971
Magnesium chloride hexahydrate	Merck	1.05833.0250
Parafin Oil	Sigma-Aldrich	76235
PEG (Polyethyleneglycol)	Sigma-Aldrich	P2263
PMSF (Phenylmethanesulfonyl fluoride)	Sigma-Aldrich	P7626
RNase	NZYTech	MB18701
SDS (Sodium dodecyl sulfate)	Sigma-Aldrich	L3771
Sodium chloride	Merck	1.06406.1000
Sodium phosphate dibasic dihydrate	Merck	1.06580.1000
Sodium phosphate monobasic dihydrate	Merck	1.06342.1000
TEMED (tetramethylethylenediamine)	Merck	T9281
Thioflavin-T	Sigma-Aldrich	T3516
Tris-HCl	Merck	1.08382.2500
Uranyl Acetate	EMS	22400

S5. References

1. Rames, M.; Yu, Y.; Ren, G. Optimized negative staining: a high-throughput protocol for examining small and asymmetric protein structure by electron microscopy. *JoVE (Journal of Visualized Experiments)* **2014**, e51087.
